# Piezoelectric Nanomaterials for Cancer Therapy: Current Research and Future Perspectives on Glioblastoma

**DOI:** 10.3390/jfb16040114

**Published:** 2025-03-24

**Authors:** Zayne Knight, Amalia Ruiz, Jacobo Elies

**Affiliations:** 1 Centre for Pharmaceutical Engineering Science, School of Pharmacy, University of Bradford, Bradford BD7 1DP, UK; 2 Institute of Cancer Therapeutics (ICT), Faculty of Life Sciences, University of Bradford, Bradford BD7 1DP, UK

**Keywords:** piezoelectric nanomaterials, cancer therapy, glioblastoma, voltage-gated ion channels, Piezo channels

## Abstract

Cancer significantly impacts human quality of life and life expectancy, with an estimated 20 million new cases and 10 million cancer-related deaths worldwide every year. Standard treatments including chemotherapy, radiotherapy, and surgical removal, for aggressive cancers, such as glioblastoma, are often ineffective in late stages. Glioblastoma, for example, is known for its poor prognosis post-diagnosis, with a median survival time of approximately 15 months. Novel therapies using local electric fields have shown anti-tumour effects in glioblastoma by disrupting mitotic spindle assembly and inhibiting cell growth. However, constant application poses risks like patient burns. Wireless stimulation via piezoelectric nanomaterials offers a safer alternative, requiring ultrasound activation to induce therapeutic effects, such as altering voltage-gated ion channel conductance by depolarising membrane potentials. This review highlights the piezoelectric mechanism, drug delivery, ion channel activation, and current technologies in cancer therapy, emphasising the need for further research to address limitations like biocompatibility in whole systems. The goal is to underscore these areas to inspire new avenues of research and overcome barriers to developing piezoelectric nanoparticle-based cancer therapies.

## 1. Nanotechnology in Cancer Treatment

Cancer remains a significant global health challenge, with high mortality rates. In 2020, breast cancer had the highest incidence, with about 2.26 million cases worldwide [1]. By 2022, there were around 20 million new cases across 36 types in 115 countries [2], and this number is projected to increase by 77% by 2050 [3]. In the UK, 2021 data show around 393,000 new cancer diagnoses and 167,000 deaths annually. Although cancer death rates are declining, the rate of decline has slowed compared to the period from 2009 to 2019 [4].

Current cancer therapies often focus on symptom management rather than a definitive cure, especially for advanced or aggressive cancers. Traditional treatments like cytotoxic chemotherapy can negatively impact patients’ quality of life due to their lack of specificity, causing side effects by targeting healthy cells along with cancerous ones [5]. This off-target interaction can lead to acute toxicity [6], necessitating higher doses and increasing patient risk. Combination therapies can mitigate these risks but require extensive development time [7,8,9]. Targeted therapies, particularly those using nanomaterials, offer promise by enhancing drug specificity, improving delivery, and overcoming physiological barriers, potentially reducing side effects and improving treatment efficiency [10].

**Figure 1:**
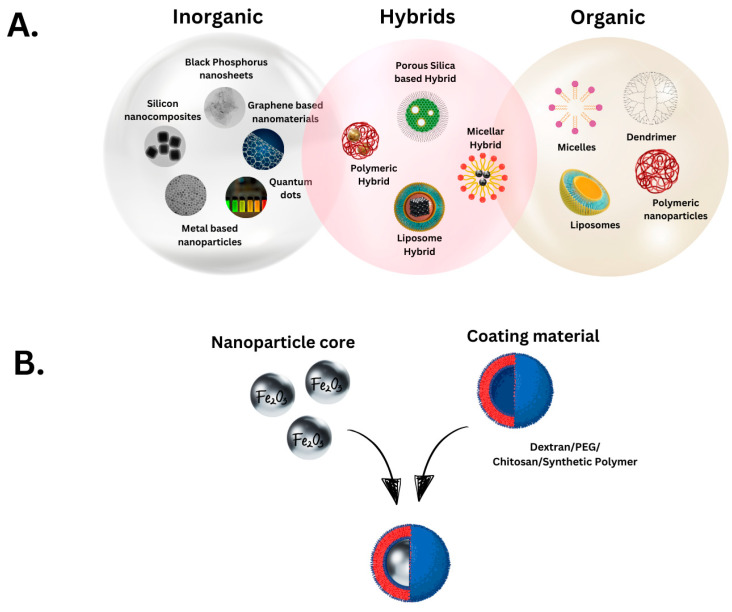
Nanomaterials classification. (**A**). Nanoparticles are broadly split into three classifications depending on their biological and engineering properties, and their use depends on the needs of the study or application. There are inorganic nanoparticles, which are made of metal or synthetic material. Organic nanoparticles are modelled on existing biological examples. Finally, hybrid nanoparticles are a mix of the two classifications and are mostly designed with a specific function in mind, unlike the other two classifications. (**B**). Piezoelectric nanoparticles are often hybrid nanomaterials (combination of inorganic and organic elements with a coating material) to ensure biocompatibility.

Piezoelectricity is a property exhibited by certain materials that generate an electric potential upon applying an external physical force. Despite advancements in cancer therapy, the potential of piezoelectricity has not been thoroughly investigated. However, in recent years, piezoelectric nanotherapy has gained traction because it can minimise harm to healthy cells while actively targeting cancer cells. The novelty lies in the encapsulation of therapeutic materials, which generate radicals that induce cell death, as demonstrated in previous studies [11]. Nanomaterials can be classified as organic or inorganic [12]; however, when delivered into a biological system, it could be argued that all nanoparticles function as hybrids to encapsulate the active agent at the desired location (Figure 1).

Nanoparticles are typically delivered within the tumour microenvironment (TME), but their delivery is affected by the body’s barriers including cell membranes, epithelial layers, endothelial barriers, and the immune system. These barriers are categorised into four main types summarising nanoparticle delivery in cancer: blood, tumour, cells, and nuclear uptake [13]. Additionally, particle size is critically important in overcoming these barriers, noting that the typical size range for nanoparticles is around 5–200 nm. Smaller nanoparticles penetrate tumour tissue better, while larger nanoparticles have better retention [14]. This indicates the importance of carefully considering particle size when designing new piezoelectric nanoparticles for delivery.

Nanoparticles exert their effects through either passive or active targeting. Passive targeting involves the accumulation of nanoparticles in neoplastic tissue. Due to the high rate of neovascularisation and large pores in cancer cells, nanoparticles can exploit these characteristics, allowing them to leak from the blood vessels [15].

Active targeting is the direct interaction between ligands and receptors, facilitated by ligands bound to the surface of nanoparticles. These ligands selectively target molecules that are characteristically overexpressed on the surface of cancer cells [16]. Various agents, such as antibodies, peptides, amino acids, vitamins, and carbohydrates, can be incorporated into nanoparticles to achieve this targeting. This approach enables the active targeting of specific changes on the cancer cell surface. A recent development in this field is the use of metal-organic frameworks (MOFs), which offer greater control over this targeting mechanism [17]. MOFs are composed of organic linkers that bind to inorganic nodes, allowing them to interact with drugs through multiple mechanisms, including van der Waals forces, hydrogen bonding, and aggregation [18]. Unlike traditional carriers, MOFs can be engineered to encapsulate drugs with precision and high encapsulation efficiency, presenting significant opportunities for advancement in oncological applications [19].

This review provides an update on piezoelectric nanotherapeutics, focusing on whether the biophysical properties of piezoelectric nanomaterials can be used to design effective nanotherapy approaches for challenging tumours like glioblastoma. It highlights the classification of piezoelectric materials, both organic and inorganic, with their potential application in biomedicine, as summarized in Table 1, emphasizing the importance of assessing biocompatibility to avoid toxicity [20].

The review also explores the principles of piezoelectricity and its potential therapeutic effects in cancer treatment, particularly for complex cancers such as glioblastoma. Additionally, it discusses future applications and perspectives, evaluates current technology, and addresses challenges in cancer therapeutics.

## 2. Piezoelectricity and Its Relevance in Physiology and Health

### 2.1. Piezoelectricity Principles and Mechanism

**Figure 2:**
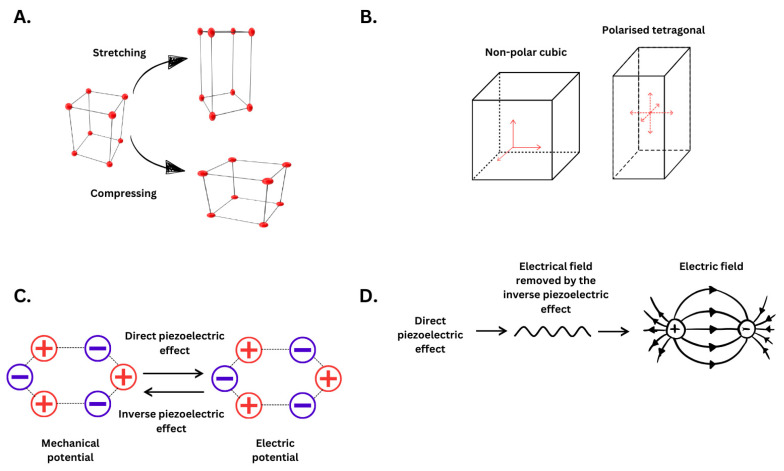
Overview of the basic piezoelectric mechanism and products of the mechanical strain. (**A**). displays a piezoelectric material under a certain mechanical stress with examples of the mechanical force that can act on the material. (**B**). displays the phase difference between activated and non-activated piezoelectric materials. The defined difference between whether a material is piezoelectric is if it is non-polar cubic or polarised tetragonal. This geometric direction displays the vibrational flow. If non-polar cubic, the centrosymmetric conformation is stable and stabilised, meaning there is no excess charge or instability between the dipoles. (**C**). shows the material in resting and the resulting dipole production with the two piezoelectric forces and how they act upon each other. Finally, (**D**). displays the production of the piezoelectric field, which is directly produced from the direct piezoelectric effect and the process of removal. Note that (**C**). applies only to a class that would be piezoelectric, not ferroelectric.

Piezoelectricity is a property of certain materials that enables them to convert mechanical stress into an electric field and vice versa. When mechanical stress is applied along the material’s polarisation direction, it generates an electric field through a direct piezoelectric effect. Conversely, when an electric field is applied, the material undergoes mechanical deformation due to the inverse piezoelectric effect, releasing mechanical energy [63]. These materials are characterised by a non-centrosymmetric crystal structure, which makes them susceptible to mechanical stimuli that initiate a change in structure to displace electron balance. A net polarisation is created due to the imbalance of the electrical charge. Of the thirty-two crystal classes, 21 are non-centrosymmetric with 20 are piezoelectric as they become polarised on the application of mechanical stress. The remaining one-point group out of the 21 does not exhibit piezoelectricity due to the combination of symmetry elements. Ten out of the 20 piezoelectric point groups display a spontaneous polarization that exists even without an applied field or stress, making them polar or pyroelectric materials. Ferroelectric materials are polar dielectrics that can reverse and reorient their spontaneous polarization when exposed to an external electric field [64,65]. This happens due to an external influence that prioritises one polarisation state over the other. In these materials, domain walls dictate separate regions with polarisation orientations. This creates a motion in the material, turning it slightly under weak electric fields [66]. Additionally, any extra external force would increase the rate of this transition and result in a quick phase as the dipoles have been further displaced by the force generated. This creates asymmetrical symmetry within the structure, producing an electrical dipole from the opposingly charged ions. As such, these materials follow certain conditions to produce an electrical field. First, the material needs to have a dielectric property, a crystal structure with no centre of symmetry, and both positive and negative charged masses, which is a structure with an ionic crystal or molecular crystal composed of ionic clusters [67]. Figure 2 displays the mechanism of action of piezoelectric materials when mechanical force is applied and the consequences of the mechanical potential, previously reviewed in [68,69].

In general, once the mechanical force is applied. The atoms are displaced from their original position within the crystal lattice, resulting in a net electrical charge through the entire structure, which creates a counterbalance from the corresponding charges.

Barium titanate (BaTiO_3_) is a well-studied ceramic non-lead-based nanoparticle and is a classic inorganic example in the research field [70]. It is defined as a perovskite-type ceramic, displaying the hallmarks of a piezoelectric material, which has been the focus of literature solely with a few combinations [33]. Some materials, like BaTiO_3_, have different conformations and produce a higher piezoelectric effect due to the different crystal phases. If we use BaTiO_3_ as an example of a piezoelectric material, it can display differing piezoelectric properties with different conformations depending on its geometric structure, such as cubic barium titanate (BTO), tetragonal unpolarised barium titanate (U-BTO), and tetragonal polarised barium titanate [71]. The morphology and size are the same; however, the electrical potentials are different due to the material’s different characteristics. BTO produces 0 mV, U-BTO produces 425 mV, and P-BTO produces 886 mV. Regarding energy potential, piezoelectric nanoparticles will follow a basic rule, and their conformation will determine the electrical output of said material. These materials are in different states; BTO produces no energy because it is non-polar and, therefore, produces no electrical field as there is no dipole movement. The polarised form induces the dipole movement and shows a displacement of the overall symmetry. The polarised orthorhombic form has a higher potential energy than polarised due to a higher distortion in the symmetry, which additionally produces more of the potential inverse piezoelectric effect. Examples include lead zirconate titanate (PZT). The highest form is that of polarised rhombohedral, resulting in the lowest symmetry without breaking the functionality. Examples of these are lead titanate ceramics and ferroelectric films [72]. Barium titanate is a good example of all four of these phases. However, Figure 2B displays a simple diagram to provide what geometry results in piezoelectricity as it can conform to the higher energy states once polarised and tetragonal.

These materials can exhibit piezoelectric effects due to mechanical stress but also change their polarisation due to the thermal expansion when encountering temperature changes. As the temperature of the material changes, positive and negative polarisation charges are present on the surface of the crystal, which is termed the pyroelectric effect [73]. The thermodynamic equilibrium is broken and established due to the change in polarisation states, which causes the absorption and desorption of chemical species. This produces a piezo-catalytic effect from producing an electrolyte-heavy environment; this, however, would be hard to apply in a biological aspect due to the heating phase [74].

### 2.2. Piezoelectricity in Biological Systems and Its Function

Electrical surface charges have a role in cells and in regulating their functions. Throughout the body, there are piezoelectric organic structures that generate electrostatic interactions, which play a critical role in biological functions such as maintaining homeostasis, protein folding, cardiac muscle contraction, and ion channel charge dependencies [68]. In addition, the scientific community has been investigating the potential for piezoelectric materials in tissue regeneration in recent years, focusing on neural and bone regeneration. Piezoelectric-specific scaffolds that match the sizes of the target damaged tissue after implantation can provide electrical stimulation to promote regeneration [75].

Recent research has revealed that many bioactive materials exhibit piezoelectric properties due to their non-centrosymmetric alignment. Peptides, for instance, demonstrate significant spatial variation and movement, enabling the formation of complex structures through various bonding mechanisms. These structures can also be organised into nanostructures that display piezoelectric properties at the macroscopic level [76]. Some proteins, such as collagen, exhibit strong potential for electrostatic interactions and are known to possess piezoelectric interactions. Helical structures like collagen can twist or stretch, producing the piezoelectric effect. However, despite these underlying principles, the resultant effect leads to a weak piezoelectric response due to a low piezoelectric coefficient [77]. Another example is elastin, an extracellular matrix protein found in various connective tissues, which provides flexibility and elasticity. Under physical stress, elastin may exhibit electromechanical responses, including potential piezoelectric behaviour, which arises from its asymmetric molecular structure [75]. At the macroscopic level, this effect manifests through the organised alignment of these proteins within tissues, such as blood vessels or lungs, where their collective response to stress integrates into measurable biomechanical behaviours. As a result, these effects could influence the dilation and contraction of blood vessels and lung tissues, although the piezoelectric properties of elastin are not yet fully understood and require further investigation [78,79]. It has also been studied that this electrical charge generated by elastin may assist in regulating the migration and proliferation of vascular smooth muscle cells while also repairing blood vessels in vascular injury [79].

The cellular targets of piezoelectric influence would be indicated to any cellular or intracellular machinery that confers a change in electrostatic potential. Many of these are widely known, but comprehensive lists include neurons, muscle cells, and most other cells, which possess some form of electrostatic potential. One notable example is the cell membrane itself. Liu and colleagues discuss these principles in detail in their comprehensive review [80]. Phosphatidylserine is negatively charged, and the authors state that electrostatic interactions govern the localisation of peripheral proteins. Finally, electrostatics are defined in two territories: membranes of early secretory pathways in weakly charged regions and membranes of late secretory pathways. All this depends on the cytosolic leaflet charge potential. Following this and the previous example of collagen as a piezoelectric material, the signals generated by collagen can be transmitted through the extracellular matrix (ECM) to the voltage-gated channels in the cell membrane [81]. This suggests that extraneous piezoelectric materials can alter overall cell dynamics and cell-cell interactions with the intended target depending on potential voltage.

## 3. Applications of Piezoelectric Nanomaterials in Cancer Therapy

The main application of piezoelectric materials when dealing with cancer is to produce an end effect of ROS generation or a desired DNA-damaging agent. However, the nanoparticle needs to be delivered and targeted to trigger an increase in ROS generation, which will result in DNA damage and reduce the side effects of the therapy. This happens due to piezo-catalysis, which produces superoxide and hydroxyl radical formation, combined with glutathione depletion. This can be targeted specifically for cancer cells if conjugated and designed, resulting in high ROS formation within the cell and, as a consequence, cancer-specific apoptosis while avoiding healthy tissues surrounding the tumour [82]. This innovative therapeutic approach makes piezo-electric nanotherapy attractive for the treatment of solid tumours.

### 3.1. Piezo-Catalysis

The generation of ROS through piezoelectric material is a specific process and involves molecular reactions downstream in biological systems. Piezoelectric materials convert mechanical energy into an electrical field, with the opposite action to be true as well. Effective charge transfer contributes to redox catalytic activity, where piezoelectric materials release electrons to catalyse redox reactions and substrates. This is termed Piezo-catalysis, and the main source of action is from ROS radical formation [83]. These oxygen-containing substances increase oxidative potentials and free radicals containing unpaired electrons. These include superoxide ions (•O_2_^−^), hydroxyl radicals (•OH), oxygen species containing singlet oxygens (^1^ΔgO_2_) and hydroperoxide (H_2_O_2_) [84].

There are two theories of piezo-catalysis, as the full mechanism is not fully established. When the piezoelectric effect takes place, it allows catalysis of redox reactions. The enriched surface of the material activated by the dipole movement will shuttle electrons to reduce oxygen molecules to O^2−^. In this state, the holes opened by stimulation allow oxidation of water molecules, which results in a net production of •OH. This is known as the electric band theory [85].

The second theory is that of the screening charge effect. This theory argues that the piezo-catalytic reaction is determined by the surface screening charges, referred to as the screening charge effect [86]. When a piezoelectric material is subject to ultrasonic vibration, the potential induces opposite polarities and can be inferred as the dipole formation. Due to this effect, equal quantities of opposing charges are attached to the surfaces. This collection of charge eventually leads to absorption and dynamic release, enabling a piezo-catalytic reaction. Under mechanical strain, this undergoes periodic absorption and release until the free energy is completely transferred through the system. The negative screening charges driving these reactions are electrons generated by dissociating absorbed water molecules on the surface or donated by absorbed •OH species [87]. The positive screening, however, is the involvement of protons. This acts the same way as negative screening, and the theory works overall by the electrons and protons influencing each other when the mechanical stress is applied.

Regarding the piezo-catalysis mechanism in tumour treatment, piezoelectric nanoparticles can generate a small electrical field with anti-tumour therapeutic effects within the targeted system. This field-generating effect is known to reduce hypoxia, a common hallmark in many cancers [88]. Consequently, this reduction in hypoxia leads to a decrease in HIFα levels, which correlates with reduced metastasis and invasion.

**Figure 3:**
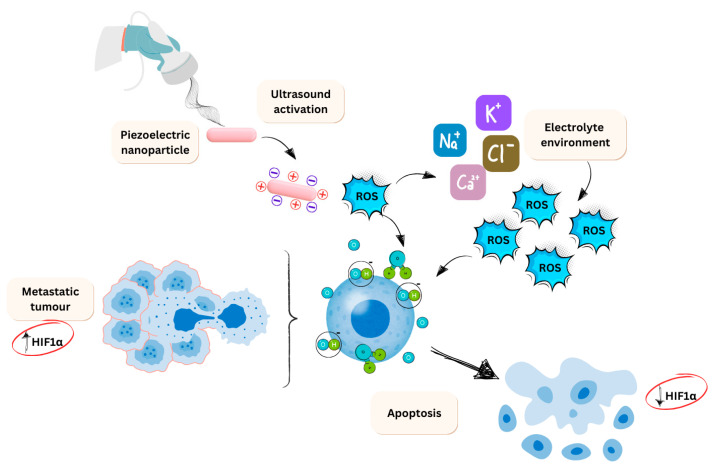
Overview of the piezo-catalysis mechanism in context to cancer cells. The figure displays that the mechanism ultimately results in apoptosis and a decrease in HIF-α. This is due to the hypoxia environment being charged to a more neutral level by the increased oxygen radicals. This process is a knock-on mechanistic approach. First, water splits to produce ROS, which affects the electrolyte environment to produce even more free radicals, which cause oxidative damage to the cells in question.

This relationship is well-documented in the literature, as a successful reduction in hypoxia typically results in lower HIFα levels, thereby diminishing metastatic and invasive potential. When a piezoelectric material is introduced and subjected to mechanical force, an imbalanced charge state is produced on its surface. This excess charge results in two redox behaviours: one directly interacts with water molecules to produce oxygen, while the other suggests that the released charges can combine with water or oxygen to generate other ROS [87]. There is a further knock-on effect on the electrolyte as the electrolyte is attracted to the material surface due to the charge. This induces water splitting to produce oxygen. Additionally, the electrolytes with opposite polarity to the bound charges are involved in the redox reaction, producing more ROS. This whole process is a step reaction and will impact the tumour microenvironment (TME). An overview of this process is displayed in Figure 3.

Typically, cancer cells produce higher ROS levels than their normal counterparts due to an imbalance of antioxidants and oxidants. This low to moderate ROS level acts as a signal transducer for cellular proliferation, migration, invasion, and angiogenesis [89]. These differences arise from molecular changes in the electron transport chain within the mitochondria. Therefore, when selectively targeted with a piezo-catalytic inducer, the increased ROS levels surpass the survival threshold, leading to cell death as cancer cells have a reduced capacity for removing harmful metabolites. In the mitochondria, complexes I and III are the primary sites of oxygen reduction, where leaking produces superoxide. In normal cells, these are converted into hydrogen peroxide by superoxide dismutase 1 (SOD1) [90]. Hydrogen peroxide is then detoxified by glutathione peroxidase (GPX) and catalase (CAT) into water and oxygen [91]. When cancer cells are suddenly exposed to piezo-catalytic materials, they exceed their capacity for ROS removal, forcing them to undergo apoptosis or autophagy to survive the influx of oxygen radicals.

Materials that are piezo-catalytic in nature and exhibit the traditional piezoelectric effect have the effect of converting mechanical stress into electrical signals. These signals stimulate cells or interfere with the biological system’s electrical field. The most notable influence is that of voltage-gated channels and biosensing, in which the piezoelectricity signal generates an effect to alter the state of signalling pathways [92]. This process does not catalyse reactions directly but promotes the production of effector molecules. This process is termed piezo-catalytic medicine as the process can indirectly be used for various applications in the medical field. Examples include connective tissue healing, remodelling of calcified tissue, and bone remodelling due to the piezoelectric nature of the bone. The exogenous charge from a piezoelectric biocompatible material can lead to osteoinduction; however, this is a relatively novel area, and the research is in its infancy [68].

Types of this therapy are already being employed in research for invasive but deep penetration treatments. An example of this is piezodynamic therapy (PzDT) investigated by Hoang and colleagues [93]. These materials are combined with ultrasound to separate the electrons and activate the piezoelectric effect, creating cytotoxic ROS redox reactions. The research involved MCF-7 breast cancer cells with a 2D PEGylated tungsten disulfide nanosheet and mouse xenografts. The investigation concluded that upon induction, the conclusion of the work generated a high volume of intracellular ROS contained in the mitochondria due to their chosen targeting molecule (FX11-LDHA inhibitor) while inducing apoptosis and suggesting that this investigation of the treatment method is successful and requires further investigation. A similar study by Gong and colleagues evaluated tetragonal barium titanate while using a combination of 5FU-based treatments for human colorectal carcinoma [32]. The study developed 3D organoids and mentioned performing “sonodynamic therapy”, but the use of a piezoelectric material suggests that there is also a lack of agreement on this terminology. However, the investigation aimed to find a way to reduce the dose-dependent amount in colorectal cancer without relying on 5FU. The work concluded that the combination was equal to high-dose drugs despite a low dose of 5FU being used when the barium titanate nanoparticle was used. However, the paper does not highlight the classification of high or low doses. The investigation concludes that the cytotoxicity of 5FU can be reduced to provide a combined therapy equal to a high dose. This suggests that pioneering piezo-catalysis with a combined therapy is the way forward for more aggressive cancers, and we should not rely on a single treatment but multiple approaches.

### 3.2. Ion Channel Activation

Ion channels are essential for life, playing diverse biological roles such as cell-cell communication, excitability, proliferation, and apoptosis. They regulate cell membrane potentials by controlling the conductance of ions like Ca^2+^, Cl^-^, K^+^, and Na^+^ [94,95,96,97]. Table 2 displays a short overview of the voltage-dependent ion channels, their cellular role and mechanism of action to summarise potential changes that piezoelectric materials may result in after application [98,99,100].

Cells have an electric potential across their plasma membrane, known as the membrane potential (Vm), which ranges from −90 to −10 mV depending on the cell type and state [101,102]. This Vm is regulated by ion channel expression in cells, the ionic composition of the extracellular milieu, and the presence of bioelectric gradients, such as an electric field (EF), within a tissue. Changes in this potential are linked to depolarisation and hyperpolarisation. Cancer cells exhibit a more positive resting membrane potential, ranging from −30 to −20 mV, compared to other body cells [103]. This characteristic aligns them more closely with rapidly dividing cells, such as embryonic and stem cells, rather than with healthy, mature cells. Additionally, ion channels are often abnormally expressed in cancer tissues and cell lines. These hallmarks of cancer cells make tumour cells targetable using piezo-electric nanomaterials.

Ion channel dysregulation in cancer impairs key cellular processes, calcium signalling, cell volume regulation, membrane potential regulation, mechanosensitivity, and microenvironment regulation [104]. Oncochannelopathies is the term used to describe the group of cancer (or tumour processes) caused by the dysfunction of ion channel subunits (or their interacting proteins), including the oncogenic roles of ion channels [105]. Blocking these ion channels can inhibit cancer cell growth, highlighting the role of bioelectrical signalling as a key feature of cancer [106]. Substantial experimental evidence demonstrates ion channels’ critical role in cellular proliferation, tumorigenesis, and metastasis. For instance, the Cav3.1 current is associated with the inhibition of proliferation and induction of apoptosis in MCF-7 human breast cancer cells [107]; and inhibition of Cav3 calcium channels decreases colorectal cancer cell proliferation [108]. In the context of glioblastoma, the downregulation of STIM1 and Orai1 results in a significant decrease in cell invasion [109]. These findings underscore the potential of targeting ion channels as a therapeutic strategy in cancer treatment.

To avoid redundancy, we will not delve into the intracellular signalling pathways associated with oncochannelopathies, as these have been extensively reviewed elsewhere [104,105,110,111,112,113]. Instead, this review will focus on three families of well-characterised ion channels: Voltage-gated Calcium Channels (VGCC), Piezo channels, and TRP channels. These ion channels are particularly relevant to this review due to their significance in glioblastoma and other types of cancer, and their modulation via piezoelectric nanomaterials. These channels possess inherent domains sensitive to changes in electrical potential or mechanical forces, which are key to their activation by piezoelectric nanomaterials. The subsequent paragraphs will summarize the biophysical properties of VGCC, Piezo channels, and TRP channels, which make them suitable targets for piezoelectric nanomaterials:

Voltage-gated Calcium Channels (VGCC or Cav channels) can be divided into two groups based on their voltage activation thresholds: high voltage-activated (HVA) and low voltage-activated (LVA) channels. Low voltage-activated Ca^2+^ channels include Cav3.1-Cav3.3 channels, also known as T-type Ca^2+^ channels. High voltage-activated Ca^2+^ channels include Cav1.1-Cav1.4 (L-type channels) and Cav2.1-Cav2.3, which comprise the P/Q, N, and R-type channels [114].

**Figure 4:**
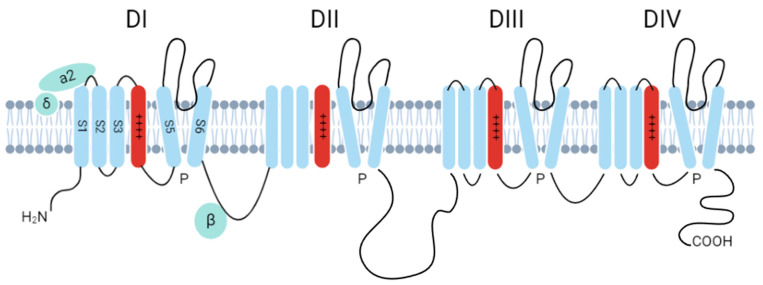
Structural domains of voltage-gated calcium channels (VGCCs). Ca_V_α1 schematic of four domains with six transmembrane units in each domain. The S4 is highlighted (red) as it contains positively charged amino acids that act as voltage sensors with the pores of the channels formed by the loop between S5 and S6. The Ca_v_β unit acts as a linker to interact with the domains and the domain linking between II–III.

The Cav family consists of 10 members, all sharing a similar pore-forming subunit (CaVα1), composed of four domains. Each domain contains six transmembrane helices (S1–S6). Specifically, S1–S4 contribute to voltage sensing, while S5 and S6, along with each domain’s P loop, form the channel’s core. The S4 segment is particularly interesting as it contains regularly spaced arginine and lysine residues that sense changes in membrane voltage (Figure 4). This sensing leads to a conformational change that transitions the channel from a deactivated state to an activated, highly permeable Ca^2+^ state [115].

Targeting the voltage-sensing domains in these channels can regulate ion channel activation. Agents such as piezoelectric nanomaterials could potentially be used to achieve this, allowing for direct control over ion influx and efflux systems. This approach could provide new therapeutic strategies for modulating cellular activities in various physiological and pathological conditions [116]. Ca^2+^ influx across the plasma membrane occurs due to membrane depolarisation. This change in membrane potential is sensed by Ca^2+^ voltage-gated channels, displaying an area of influence where piezoelectric materials can affect the fate of cells by interfering with the ion channel domains sensitive to the changes in membrane potential.

There is substantial evidence supporting the role of VGCC in cancer. For instance, Cav1.2 and Cav1.3 channels have a direct effect on tumour cells, and blocking these currents reduces cell proliferation [117,118,119]. Additionally, blockade of L-type Ca current attenuates doxorubicin-induced cardiomyocyte apoptosis via suppression of the CaMKII-NF-κB pathway [120]. This suggests that nano-piezomaterials could potentially reduce the toxicity of chemotherapeutic drugs. These research findings demonstrate the significant role of piezosensitive ion channels in cancer. This review will focus on glioblastoma, where NMDA receptor dysregulation is common. NMDA receptors are involved not only in excitotoxic cell death but also in pathological cellular proliferation, contributing to the invasion and proliferation of glioblastoma cells and other types of cancer [121,122,123,124].

Targeting VGCCs that are downregulated in cancer could lead to new research opportunities. However, most current studies focus on upregulated channels. For example, voltage-gated sodium ion channels (VGSCs) are often overexpressed in many cancer types, promoting cell invasion and metastasis [125]. Another approach could be to overload these channels beyond their depolarisation limit. Calcium channels have received the most attention in this context. Alterations in ion channel expression can initiate, proliferate, and metastasise cancer cells [126]. Key pathways affected by these changes include mitogen-activated protein kinase (MAPK), ERK, JNK, Wnt/ß-catenin, PI3K/Akt, Notch, and the Rac and Rho pathways [127]. Studies have shown that changes in Ca^2+^ and K^+^ ion channels are directly involved in cell migration and EGFR signalling.

Calcium is particularly important because it plays a role in cancer initiation through epithelial-mesenchymal transitions (EMT). Targeting Ca^2+^ channels could prevent cancer progression. For instance, PMCA4 has been shown to inhibit this pathway in gastric cancer. Further details on ion channel changes and their intracellular pathways in cancer are available in other reviews [128].

The discovery of new families of mechanically activated ion channels, such as PIEZOs, which play crucial physiological roles in mammals [129,130,131,132,133] has opened new avenues of inquiry into the roles of mechano-transduction in human health and disease and the development of novel therapeutic strategies aiming to modulate the activity of these ion channels.

Changes in Piezo1 have been identified in various cancers, with overexpression promoting invasion and metastasis in gastric cancers [134]. Piezo1 is also highly unregulated in thyroid and breast cancers, making it a promising target for a wide range of cancers. If Piezo1 ion channels could be targeted with piezoelectric biocompatible materials, it might have a therapeutic effect or provide insights into how these materials affect tumour progression.

Higher expression of Piezo1 is a hallmark of cancer cells. Knocking down caveolin1 or reducing cholesterol content inhibits Piezo1 expression [135]. Piezo 1 also promotes single-cell migration by modulating adhesion factors, notably the cytoskeleton protein F-actin, and forming invadopodia through calcium influx. This calcium influx affects downstream effectors such as ERK and Src. Additionally, elevated Piezo1 levels inhibit fibroblast formation, reducing adhesion with cadherins. In the context of piezoelectric nanoparticles, targeting Piezo1 mRNA could enhance the delivery effectiveness of an inhibitor if designed correctly. This approach could provide a combination therapy that uses an electrical field to depolarise any voltage-sensitive machinery.

The transient receptor potential (TRP) family of channels plays a critical role in various biological processes, including cell cycle regulation and cancer progression. Many TRP family members function as mechanosensory channels, making the TRP family a promising therapeutic target. Their involvement in multiple stages of cancer development and metastasis highlights their potential for therapeutic intervention [136,137].

TRPV6, for example, is well-documented for its high expression in various cancers, including those of the prostate, breast, colon, and oesophagus. The high expression is associated with metastasis and chemotherapy resistance [138]. Another example of the TRP family, TRPA1, is expressed in nociceptor neurons and acts as a chemosensory receptor for noxious compounds. Berrout and colleagues [139] investigated TRPA1 and concluded that it serves as a prognostic marker in cancer. Their research suggests that FGFR2 binds to TRPA1 to form a complex facilitated by ankyrin, which binds TRPA1 to the C-terminal proline-rich region of FGFR2. This interaction inhibits the channel, leading to a conformational change that promotes the proliferation and invasion of lung adenocarcinoma (LUAD).

However, further research is needed to determine if these findings can be translated into other cancer types. The study also implies that astrocytes target infiltrating LUAD cells by depleting TRPA1-expressing exosomal miRNA, thereby interrupting FGFR2 binding. Additional mechanistic insights are required to understand these interactions fully. The piezoelectric effect may influence both TRPV6 and TRPA1 channels, offering potential therapeutic applications alongside inhibitors or exciters. However, these options remain unexplored and can only be inferred from the current understanding of their mechanisms of action.

#### 3.2.1. Evading Apoptosis

**Figure 5:**
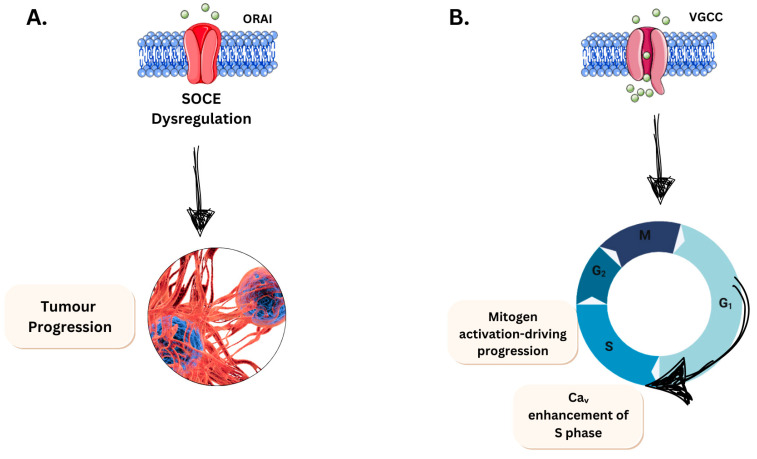
Scheme summarising main calcium-handling events on cancer cells. (**A**). Reduction of SOCE is associated with apoptotic-resistant phenotype and subsequent tumour progression. (**B**). Increased Ca^2+^ influx via VGCC induces cell proliferation via promotion of S phase. These ion channels are ideal targets in piezoelectric nanotechnology.

Calcium ion influx is required for cell cycle progression, and reducing the levels of extracellular Ca^2+^ terminates progression via the G1 phase. Halting cells at the G1/S phase boundary and mitogens induce calcium entry pathways to drive cellular progression through the cell cycle. Of the voltage-gated Ca^2+^ channels (VGCC), Cav3 channels are stated to be expressed in many cancer cell lines, and the increased expression exhibits increased S phase activity, which is inferred to result in higher DNA synthesis [140]. One major hallmark of cancer is evasion of apoptosis, in which cancer cells must utilise mechanisms to reduce Ca^2+^ influx either by altering the signalling pathways or downregulating the calcium-permeable channels (Figure 5). Apoptosis-resistant phenotypes are characterised by a reduction in store-operated calcium entry (SOCE), which prevents calcium ion overload from pro-apoptotic stimuli. The effect reduces the efficiency of mitochondrial and cytoplasm apoptotic pathways. A study performed by Liu and colleagues [141] investigated glioblastoma survival with the introduction of amlodipine and discovered, on its introduction, activated Ca^2+^ entry. This further enhances SOCE and inhibits YAP/TAZ in glioblastoma by activating Lat1/2 kinases. The conclusion from this work indicated that activating SOCE to suppress YAP/TAZ signalling to interfere with extracellular calcium L-type channels may be a reliable focus on reducing glioblastoma clinically and other cancers. Other examples of bioelectric control modulated by Orai/SOCE have been described in breast [142] and cervical [143] cancer growth and metastasis [128,144].

#### 3.2.2. Limitless Growth Potential

**Figure 6:**
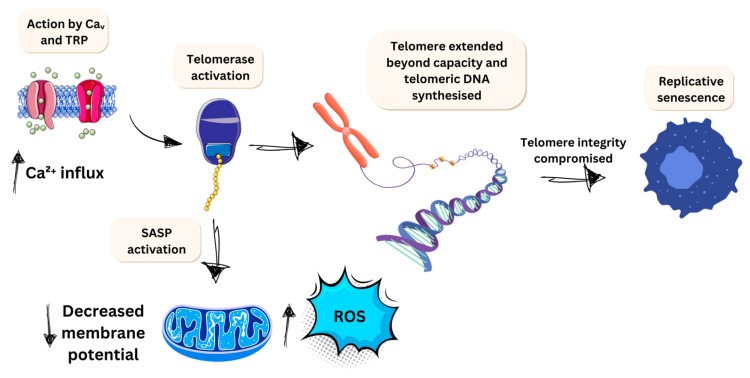
Scheme of somatic replicative senescence and oncogenic senescence in cancer cells. The figure illustrates key mechanisms that contribute to the limitless potential of cancer cells, highlighting the role of TRP and Cav channels in Ca^2+^ release.

Focusing on a different hallmark to provide scope to voltage-gated ion channels is important to downstream mechanisms. The limitless replicative potential is a hallmark negatively influenced by Ca^2+^ ions. Most somatic cells have a set limit to their replicative growth, known as the Hayflick limit, which varies by cell type [145]. This potential is determined by telomeres, which consist of several thousand repeats of 6 bp sequences that protect chromosome integrity. However, with each division, telomere shortens by 50–100 bp, depending on the cell type, eventually leaving the chromosome unprotected as the repeats are exhausted. This leads to replicative senescence in cells. Cancer cells, however, can extend this limit through the action of telomerase, which stabilises or synthesises telomeric DNA [146]. This extension of instability is facilitated by the influx of calcium through VGCC or TRP channels, promoting telomerase activation (Figure 6). This elevation of intracellular calcium has been investigated and confirmed in other types of senescence. Notably, Martin and Bernard [147] describe that the Cav1.3 L-type channel prevents a rise in rotenone-induced senescence in neuroblastoma SH-SH5Y cells. This intracellular increase also promotes the senescence-associated secretory phenotype (SASP), which has a knock-on effect on local inflammation. It promotes increased mitochondrial ROS via a drop in mitochondrial membrane potential. This enhances ROS production, adding to the potential for mutagenic damage in already altered DNA strands. During oncogenic senescence and telomere shortening, calcium is released from endoplasmic reticulum stores via the PLC/IP3/IP3R pathway [148]. This increased calcium is sustained for senescence, indicating that VGCC and activated machinery play a role in controlling this hallmark’s progression in cancer types. This effect is particularly evident in cells that are not meant to divide and have short base repeats, such as neural cancers.

These areas are key for the potential expansion of research, as many studies give the mechanism of action of these ion channels in cancer development. Karska et al. [135] review this in detail, highlighting common cancers and the expression of ion channels closely associated with enhancing tumorigenesis. Mechanosensitive ion channels, such as Piezo and TRP families, are linked to mutations that facilitate tumour progression. Specifically, Piezo channels are activated by mechanical stretching of the cell membrane, while TRP channels are activated by changes in temperature, pressure or chemical environment [149,150].

### 3.3. Drug Delivery

The uniqueness of piezoelectric technologies in cancer is the application of wireless electrical stimulation, which has clear harmful effects and is not always readily accessible to a patient. Piezoelectric materials can be activated by ultrasound-based external pressure, which results in electrical stimulation by the direct piezoelectric effect [64]. This means the electrical stimulation can be directed to a specific tissue or cellular environment depending on the penetrating power that remotely generates an electrical charge. However, mechanical stimulation can include ultrasound, tide, water flow, wind, agitation, mechanical stirring, ball-milling, and vortex [151]. However, when critically appraising the mechanical stimulation sources, ultrasound is the most suited for biological systems unless activated beforehand and placed into the patient through a different delivery system. The ultrasound wave is a mechanical wave with a frequency of >20 kHz, which needs a medium to travel. On initiation, it produces cavitation bubbles that provide mechanical pressure to these nanoparticles. Ultrasound can penetrate up to 5 cm in depth through tissue, and one unique ability of this technique, unlike the other stressors used for a direct piezoelectric effect, is that ultrasound can modulate the mechanical stress, manipulating the piezoelectric effect [152]. However, a more recent paper argues that this can be a depth of 4.5 cm by implanting membranes at different depths of porcine tissue, generating a high voltage of 8.22 V [153] This provides a medium for targeting cancers specifically by the area instead of cytotoxic chemotherapy or general radiotherapy methods, reducing adverse effects. The main clear advantage of piezoelectric nanoparticles regarding drug delivery is that the surface charge of the material regulates the cell membrane permeability. This has the additional ability to activate immune pathways, which are discussed further in Section 3.5 [154]. Interestingly, piezoelectric nanomaterials can be integrated with other delivery systems, such as polymers and hydrogels, to enhance their biocompatibility and efficiency [155,156]. However, further research is required to demonstrate their translation into clinical practice.

### 3.4. Electrodes for Tumour Treating Fields

Tumour treating fields (TTFields) are a novel cancer treatment modality that uses alternating electric fields of intermediate frequency (100–500 kHz) and low-intensity electric fields (1–3 V/cm) to disrupt cell division [157]. The frequency range could be selected based on the cancer type and sensitivity [158]. TTFields are typically applied by attaching electrodes to the target area to generate an electric field, with the frequency adjusted accordingly. This therapy is approved for glioblastoma patients at a frequency of 200 kHz [159]. The current used to create the field generates heat, and exceeding certain thresholds can be harmful. The reported maximum safe current density is 31 mA/cm^2^ RMS, while the threshold for causing burns is 100 mA/cm^2^ RMS. The skin can sustain thermal injuries at temperatures above 41 °C, with a critical injury threshold at 44 °C. Foster et al. provided a detailed analysis of heat distribution, skin type, thermal frequency, and exposure time to assess these risks [160].

Although the exact mechanism is not fully understood, TTFields are effective on mitotic cells because they target the mitotic spindle and microtubules [161,162]. Mitotic cells are highly polar, making them susceptible to externally applied electric fields, which disrupt spindle formation and activate the spindle assembly checkpoint (SAC). This leads to apoptosis, similar to the mechanism of action of microtubule-targeting agents, such as vinca alkaloids, taxanes, and epothilones. Also, TTfields can disrupt subcellular organelles, promote autophagy, and perturb cell membranes of cancer cells [163].

An alternative hypothesis suggests that the hourglass shape formed during anaphase causes the electric field to push charges and dipoles towards the cleavage furrow [164]. The non-uniform electrical field interferes with this process, extending the mitosis phase. Lee and collaborators describe this as dipole alignment by TTFields, which inhibits tubulin polymerisation, ultimately resulting in metaphase arrest due to improper spindle formation [165]. This further leads to abnormal chromosome segregation and cell death.

**Figure 7:**
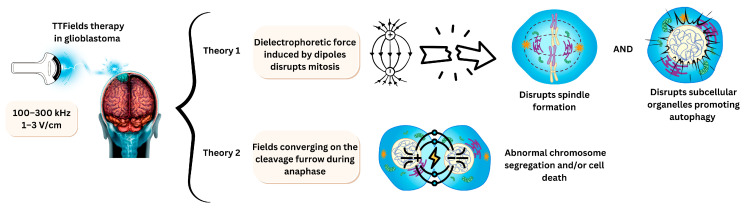
Overview of current theories explaining tumour treating fields in glioblastoma. These two individual theories focus on tubulin and mitotic spindle disruption resulting in cell death.

TTFields can also lead to replication stress, as demonstrated by Karanam and colleagues [166]. Their study shows that the effect of TTFields can be measured by observing polymerase progression. TTFields directly induce replication stress by decreasing the speed of DNA replication and increasing the duration of R-loop formation compared to treatments without TTFields. Figure 7 summarises these two theories, clarifying a key point of reference for the mechanism of action [159,164,167].

### 3.5. Nano-Piezoelectric Immunotherapy

Alongside the potential to cause cell death in cancer cells via radical formation, piezoelectric materials also have been shown to play a role in tumour immunotherapy. Piezoelectric materials are known to activate the immune system by modulating electrical signals, which directly alter the behaviour of immune cells via electrical mediation [73].

**Figure 8:**
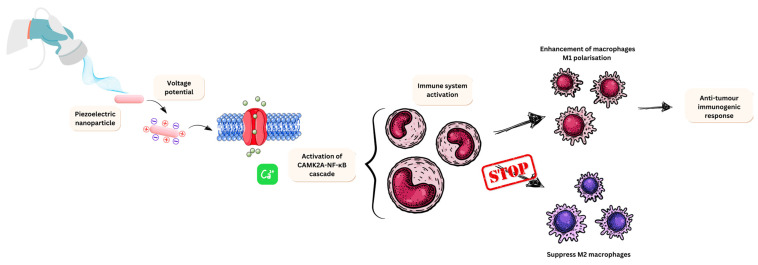
Macrophage activation upon introduction of the piezoelectric nanoparticle. This scheme illustrates the M1 macrophage polarisation pathway. The electrical stimulation via the electrical field produced as a knock-on effect by the ultrasound creates a pro-inflammatory effect in the host. This activates a cascade to inhibit M2 macrophages, mitigating the inflammatory response while enhancing the M2 response. An action needed greatly for cancer-specific immunity and recognition.

A study performed by Kong et al. [168] demonstrated that localised charge enhances the M1 polarisation of macrophages. The study continued to explain that ultrasounds induced a voltage potential, resulting in an influx of Ca^2+^. This then activated the Ca^2+^-CAMK2A-NF-κB cascade, which is involved in survival and death and relies on intraocular Ca^2+^ to be activated [120]. This resulted in a proinflammatory response from M1 macrophages. This piezoelectric response both enhances M1 polarisation and inhibits M2 macrophages. M1 macrophages overexpress CD80, CD86, and CD16/33. These create pro-inflammatory cytokines, which drive immune recruitment and, in this context, a tumour immune response [169]. M2 macrophages express anti-inflammatory factors (IL-10), CCL17, and CCL22. These factors all recruit T-regs, which suppress the immune response by recruiting CD4^+^, which subsequently suppresses CD8^+^ cytotoxic T lymphocyte function but aids in tissue repair and homeostasis [170]. Overall, the piezoelectric response causes a hike in M1 macrophages, which promote immune responses but suppress M2 macrophages, which would promote a tumour microenvironment and further suppress angiogenesis. An overview of this mechanism of action can be observed in Figure 8.

**Figure 9:**
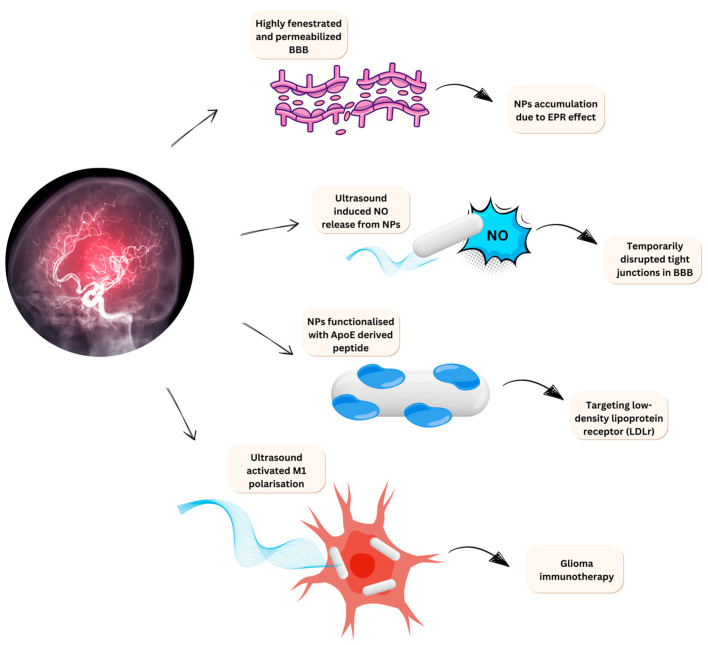
Mechanisms of Piezoelectric Nanoparticles in Targeted Glioblastoma Treatment. From top to bottom: (1) Accumulation of nanoparticles (NP) due to the enhanced permeability and retention (EPR) effect; (2) Disruption of BBB via NP-dependent nitric oxide (NO) release; (3) Targeting low-density lipoprotein receptors (LDLr); and (4) Ultrasound-activated M1 polarisation, in which BaTiO_3_@Glu NPs increase MHC 2 response, leading to an enhanced CD4+ lymphocytes-dependent antigen-specific immune response.

Another example of nano-piezoelectric immunotherapy was developed by Miao et al. [171]. This study utilised BaTiO_3_ conjugated glucan nanoparticles (BaTiO_3_@Glu NPs) and focused on ROS production. Glucan was used as an immunological adjuvant to enhance the immune response. The study specifically examined the efficacy of BaTiO_3_@Glu NPs in RAW264.7 macrophages and colon cancer cells, demonstrating findings consistent with those from previous studies [168]. Nanoparticle uptake following ultrasound stress led to increased levels of proinflammatory cytokines, including tumour necrosis factor-alpha (TNF-α) and interleukin 12 (IL-12). Additionally, there was an increase in CD86 and major histocompatibility complex class 2 (MHC II) biomarkers, indicating that glucan contributed to the observed MHC II and CD86 responses. This study provides insights into enhancing the nano-piezoelectric immunotherapeutic effect by activating macrophage responses through Ca^2+^ influx and using glucan as an immunological adjuvant to boost the existing immunological effects of M1 macrophages. This piezoelectric mechanism of action is depicted in Figure 9, together with other three potential mechanisms by which piezoelectric nanomaterials can be designed as a therapeutic tool in glioblastoma.

Based on the studies discussed above, Table 3 summarises the main properties, size ranges, advantages, and limitations of key piezoelectric materials used in cancer therapy.

### 3.6. Piezoelectric Nanomaterials for Glioblastoma Treatment

Glioblastoma (GBM) is one of the most prevalent malignant brain tumours in adults, accounting for 30–40% of all such cases [186]. Current management of GBM involves maximal safe surgical resection followed by radiation and chemotherapy. However, the prognosis remains poor, with a median survival rate of 14.6 months from diagnosis [187]. This poor outcome is primarily due to resistance to existing therapies, which leads to recurrence. Consistently, there is a pressing need for novel therapeutic strategies to manage glioblastoma recurrence.

It has been reported that systemically administrated piezoelectric nanoparticles were used to cross BBB and ultrasound-driven dopamine release to activate dopaminergic neuron like cells [188]. This novel report discusses the ability of piezoelectric nanoparticles to cross the BBB via nitric oxide release to temporarily disrupt the tight junctions in the BBB and favour brain penetration. A similar mechanism can be employed to design an ultrasound-responsive, on-demand drug delivery system for the treatment of GBM.

In an interesting study, polymeric piezoelectric nanoparticles were explored as a novel immunotherapy agent to reprogram the glioma-associated microglia from a tumour-supportive M2 phenotype to an antitumour M1 phenotype. Here, they used the wireless electrical signal produced by the camouflaged piezoelectric nanoparticles (piezoelectric nanoparticles coated with GBM cell membrane extracts) to locally generate electrical cues on GAM membranes, activating their M1 phenotype and ultimately triggering a promising anticancer activity against GBM (Miao et al. [171]. Although current glioblastoma treatments face challenges like drug resistance, poor blood-brain barrier (BBB) permeability, and lack of tumour specificity, some pioneering studies have developed cationic fluorescent anticancer agents that can penetrate the BBB and induce paraptosis and ferroptosis in GBM cells by targeting mitochondria and causing non-apoptotic cell death [189].

## 4. Advantages and Current Challenges

The advantages of piezoelectric nanomaterials in cancer therapy are numerous. They can be engineered to target cancer cells, reducing harm to healthy tissues specifically. As described above, they can convert mechanical energy, such as ultrasound vibrations, into electrical energy. This property can be harnessed to generate reactive oxygen species (ROS) that can kill cancer cells, offering a non-invasive treatment option. Piezoelectric nanomaterials can be combined with other treatments like chemotherapy to enhance effectiveness. They also present the potential advantages of helping to overcome resistance in cancer cells, improving treatment success, and improving tumour visualization, aiding in diagnosis and monitoring.

The primary challenge in using piezoelectric materials for cancer treatment is the toxicology of the materials currently in use. As shown in Table 1, biocompatibility is critical for the therapeutic application of piezoelectric materials. For instance, lead-based materials are unlikely to be used unless a comprehensive toxicological profile is established. Many studies have investigated the treatment of cancers using piezoelectric materials like barium titanate. Ahamed et al. [173] observed cytotoxicity in lung cancer cells (A549 cells) due to oxidative stress and free radical production. Similar studies have been conducted on other cancers and the development of piezoelectric materials. For example, Srinivasa Rao et al. [190] investigated the use of a drug delivery system with a piezoelectric mechanism as the basis for micropump delivery into a patient’s body. Despite these advancements, most studies are conducted in closed systems or on individual cell lines. While these studies provide valuable insights into the behaviour of piezoelectric materials in controlled environments, they do not demonstrate how these materials interact with multiple systems. They also fail to provide comprehensive toxicological data on whole systems, including the antitumor effects and dose-response relationships. This indicates that further research is needed to scale up studies with 3D and animal models to obtain detailed toxicology reports.

### 4.1. Challenges of Using Piezoelectric Nanomaterials in Glioblastoma

Glioblastoma is the most common primary brain tumour, classified as a grade IV glioma, and accounts for 60–70% of malignant brain tumours. The life expectancy for patients is approximately 12–18 months [191]. The current standard of treatment includes surgical resection followed by radiotherapy and temozolomide. Glioblastoma persists despite treatment because it infiltrates deep into the brain, making additional resection impossible without fatal consequences [192]. This highlights the need for alternative treatments, such as using piezoelectric nanoparticles to target aberrant voltage-gated ion channels. However, challenges such as the blood-brain barrier (BBB) and the need for biocompatible nanoparticles must be addressed.

The BBB, composed of microvascular endothelial cells, acts as a barrier while communicating with astrocytes and pericytes. This anatomical feature creates tight junctions between endothelial cells, forming pores with diameters of 1.4 nm–1.8 nm [193]. Therefore, piezoelectric nanoparticles must be small enough to pass through these tight junctions and navigate the unique immune environment of the BBB and brain. Modulating the BBB is a significant task, with studies focusing on using tight junction modulating agents, drug efflux transport inhibitors, and ultrasound. Developing these methods further could allow the conjugation of piezoelectric nanoparticles, enabling comparisons with standard nanoparticles like gold to explore their therapeutic potential.

### 4.2. Therapeutic Benefit of Using Tumour-Treating Fields

Many tumour types, such as glioblastoma, are difficult to treat and have a very poor prognosis. Targeting these areas specifically is a significant challenge. Current treatment methods often produce side effects that are detrimental to the patient. An interesting follow-up clinical study demonstrated that tumour-treating fields (TTFields) have been applied to glioblastoma patients, however, the average survival remains under two years, with a maximum extension of one additional year (Stupp et al. [194]. Olatunji and colleagues investigated the outcome of Stupp and collaborators’ investigation of using the tumour-treating field in current glioblastoma therapy [195]. Combining the standard treatment of glioblastoma and tumour treating fields, the effect was an extension of median survival by 4.9 months. They also noted that this increased the quality of life for the patient with no substantial rise in the rate of adverse effects. This was a randomised trial consisting of 695 patients, with those patients already having completed initial radiochemotherapy. While this combined therapy has its limitations, targeting the brain via a portable device does confirm clinical success. It proves that for future work, the use of TTFields improves therapeutic outcomes in glioblastoma patients. This principle is focused on glioblastoma; however, it may be a useful therapeutic tool for other persistent cancers if piezoelectric nanoparticles are developed for deep tissues.

Using piezoelectric nanomaterials offers a novel approach to addressing the challenge of treating cancer as a whole rather than targeting specific tumour types. Yu et al. [196] describe the benefits of piezocatalysis in detail, emphasising how these benefits optimise current targeting strategies. Nanoparticles smaller than 100 nm suggest favourable endocytic cell uptake. Additionally, 1D and 2D structures exhibit a greater piezoelectric response than 3D morphology. Combining these effects with conjugation and drug delivery is likely to produce a more significant therapeutic response.

This treatment method also offers a safer alternative to using cytotoxic agents. Marino and colleagues suggest this perceived safety in breast cancer cells, noting that despite being wire-based, the effects simulate mobile conditions similarly [172]. They found that cancer cells can be countered by applying electrical stimulation to develop multifunctional nanosystems. Their study concluded that electrical stimulation enhanced the cytotoxic effects of chemotherapy drugs, potentially allowing for lower dose concentrations and reducing harmful effects for patients.

## 5. Conclusions and Future Perspectives

This review highlights the promising potential of piezoelectric nanomaterials as a novel treatment method for various solid cancers. As an emerging area of research, the application of piezoelectric nanomaterials is still in its infancy and much remains to be explored. The benefits of this approach in the context of patient treatment are not yet fully understood. However, the potential advantages are significant, including the possibility of reducing or even eliminating the need for cytotoxic drugs. Additionally, piezoelectric nanomaterials could be combined with other treatment modalities, such as photothermal therapy, magnetic hyperthermia, chemotherapy and/or radiotherapy to enhance therapeutic outcomes.

Developing piezoelectric nanomaterials for cancer therapy could lead to wide-ranging applications, potentially increasing survival rates for tumour types currently considered untreatable with standard therapies. Also, combination therapies such as piezo-photodynamic therapy and piezo-magnetic hyperthermia hold significant potential in cancer treatment. New avenues of research could be opened to explore the application of piezoelectric materials as biosensors for the early detection of cancer by measuring the fundamental frequency variations of the piezoelectric resonator. The physicochemical properties of these materials also support their application in precision medicine approaches using label-free and cost-effective solutions. Their electronic properties can be further explored to design point-of-care devices monitoring biomarkers beyond blood or urine fluids for specific healthcare needs in real time.

The nanoscale piezoelectric effect offers a unique mechanism that could revolutionise the field of cancer therapeutics. However, several challenges remain, particularly in understanding the delivery, targeting, and mechanisms of action within cellular models.

Future research should address these challenges to define the practical limitations and optimise the use of piezoelectric nanomaterials in clinical settings. Investigations into the precise mechanisms of action, effective delivery systems, and targeted approaches will be crucial. Additionally, exploring the combination of piezoelectric nanomaterials with existing therapies could provide synergistic effects, further enhancing their therapeutic potential. Finally, a safe-by-design approach in developing these materials will ensure a faster translation into the clinic, a reduction of human and environmental risks and an improved socioeconomic impact. With this approach, the development of biocompatible and biodegradable materials can also contribute to the development of wearable point-of-care devices that can monitor patient biomarkers for early detection or the progression of cancer in real time, significantly impacting patient management by clinicians.

In conclusion, while the use of piezoelectric nanomaterials in cancer treatment is still in the early stages of development, the potential benefits are substantial. Continued research and development in this area could lead to significant advancements in cancer therapy, offering new hope for patients with difficult-to-treat tumours.

## Figures and Tables

**Table 1:** Piezoelectric-specific materials and their applications in biomedicine. Many materials are piezoelectric and have electrostatic interactions that generate small electric fields for biological and engineering applications. The table provides an overview of the classification that may be utilised for purposes in the biomedical or biological field.

Type of Nanomaterial		Specific Material		Potential Application in Biomedicine	Refs.
Synthetic Materials	Inorganic ceramics	Lead-based	Lead titanate	Electrochemical detection of nitrous oxide	[21,22,23]
			Lead lanthanum zirconate	Electro-optic applications. Ultrasound imaging, reduction in aperture, wireless device applications, stabilising additive.	[24,25]
			Lead zirconate titanate	Ceramic resonator for cancer biomarker detection and wireless monitoring applications.	[26,27,28]
			Lead magnesium niobate	Medical ultrasound imaging (biomedical measuring).	[29,30]
		Lead-free	Barium titanate	Tissue engineering, bioelectrical sensing, cancer therapy, drug delivery, gene engineering.	[31,32,33]
			Lithium tantalate	Infrared detection and acoustic wave devices, glass and or bone engineering.	[34,35]
			Iron oxide	Drug delivery, iron supplementation, cancer therapy, gene carriers, macrophage polarisation.	[36,37,38]
			Zinc ferrite	Considered non-toxic. Cancer therapy (thermal ablation), antibacterial/antifungal, and inorganic/organic pollutant removal.	[39,40,41]
	Organic polymers	Poly (vinylidene difluoride)		Multifilament for vascular grafts, ligaments, and corneas. Also, drug and diffusion media. It can also be used as an encapsulation material.	[42,43,44]
		Nylon		Hybridised materials such as skin dressings and composites for piezoelectric bone stimulation.	[45,46]
		Polyacrylonitrile		Hybridisation with metallic materials as an antibacterial agent. It could be used in wound healing and prevention. Hydrophilic and highly elastic material.	[47,48]
		Cellulose acetate		Tissue engineering as a primary scaffold or scaffold structures to osteoblast/fibroblast anchoring.	[49,50]
	Polymer composites	PVDF/PZT		It could be used for portable devices, mobile imaging, and electrical storage.	[51]
		PVDF/ZnO		Organic electro-spun piezoelectric nanofibers. Self-powered sensors to monitor the cardiovascular walls of the heart.	[52]
		PVDF/BT NPs		Increased electrical wireless stimulation in bone scaffolds for wound healing and oestogeneration.	[53]
		PVDF/MOS_2_		Next-generation sensors for wireless biomedical applications.	[54]
Natural Materials		Quartz crystals		Used in quartz crystal microbalance systems, which are used as wide-range biosensors.	[50,55]
		Rochelle salt		To be developed as a 3D composite for prosthetics and smart sensing impact-prone professions (self-generative material).	[56,57]
		Silk		Tissue engineering, cell coating, drug delivery, microfluidics, and formation of composite biomaterials.	[58,59]
		Bone		An inherent piezoelectric material. Piezoelectrics contribute towards the promotion of regeneration and repair.	[60,61,62]

**Table 2:** Short overview of relevant ion channels, detailing their cellular roles and mechanism of action by which piezoelectric nanomaterials can modulate ion channel activity.

Ion Channel Class	Cellular Role	Mechanism of Action
Voltage-gated potassium channels	Regulate excitability and control the action potential waveform. Help with the secretion of hormones.	Activity regulated by calcium, voltage, and neurotransmitters
Voltage-gated chloride channels	Cell volume regulation, salt transport, and importantly, acidification of intracellular and extracellular components with cell cycle signalling.	Anion-selective and activated by intracellular calcium.
Acid-sensing ion channels	Permeable by Na^+^ and serves to detect extracellular PH from neuronal response.	Gated by membrane depolarisation by transmembrane PH gradient
Volage-gated sodium channels	Allow movement of electrically charged particles. Dysfunctions of these, change the charge potentials of cells	Gated by depolarisation, and rapid inactivation, and are the first channels to respond to changes in voltage.
Voltage-gated calcium channels	Present in the membrane of highly excitable cells. Works as a secondary messenger with many functions across the cell.	Form hetero-oligomeric complexes with the α1 subunit providing extracellular binding sites. Ca_v_3 type (T types) are low voltage-activated, unlike L types.
Piezo channels	Expressed in mechanically sensitive cells. Allow Ca^2+^ influx and are observed in a variety of endothelial cells to sense physiological shear stress.	Allow the Ca^2+^ influx in response to external force. The main function and action of this channel is that of mechanical transduction.

**Table 3:** Summary of key piezoelectric materials for cancer therapy, highlighting their main properties, size ranges, advantages, and limitations.

Piezoelectric Nanomaterial	Size Range	Advantage	Limitation	Reference
Barium Titanate nanoparticles	50–300 nm	-High Piezoelectric coefficient -Can be surface functionalised	-Possible inflammatory response	[155,172,173,174,175,176,177]
Zinc Oxide nanoparticles	30–150 nm	-Biocompatible and biodegradable -Good piezoelectric responses	-Potential toxicity at high concentration	[155,178,179,180]
Polyvinylidene Fluoride (PVDF) nanofibers and nanoparticles	20–100 nm	-Flexible and biocompatible	-Limited piezoelectric response compared to inorganic materials	[181,182]
Polyvinylidene fluoride-trifluoroethylene (PVDF-TrFE) micro particles	2–6 µm	-Flexible, high piezoelectric output,	- Requires specific processing to enhance piezoelectric response	[183,184,185]

## Data Availability

No new data were created or analyzed in this study. Data sharing is not applicable to this article.
